# “Lazarus Response” to Olaparib in a Virtually Chemonaive Breast Cancer Patient Carrying Gross BRCA2 Gene Deletion

**DOI:** 10.7759/cureus.2150

**Published:** 2018-02-04

**Authors:** Vladimir M Moiseyenko, Vyacheslav A Chubenko, Fedor V Moiseyenko, Lyudmila A Zagorskaya, Yuliya A Zaytseva, Nataliya E Gesha, Evgeny N Zykov, Valeriya I Ni, Elena V Preobrazhenskaya, Anna P Sokolenko, Evgeny N Imyanitov

**Affiliations:** 1 Director, City Cancer Center, Saint Petersburg; 2 Department of Chemotherapy, City Cancer Center, Saint Petersburg; 3 Laboratory of Nuclear Diagnostics, City Cancer Center, Saint Petersburg; 4 Department of Tumor Growth Biology, N.N. Petrov Institute of Oncology, Saint Petersburg

**Keywords:** brca2, gross rearrangements, olaparib, parp inhibitors, breast cancer

## Abstract

This report describes an estrogen receptor-positive breast cancer patient, who relapsed at two and a half years after the completion of adjuvant chemotherapy while being on the aromatase inhibition. Based on the clinical evidence for potential sensitivity of the tumor to hormone ablation, everolimus was added to continuing exemestane treatment. Oral chemotherapy was administered at further disease progression, however, it lasted only for 10 days due to rapidly deteriorating condition of the patient. BRCA test was performed just before the failure of endocrine therapy and revealed a gross deletion within BRCA2 gene. Since the patient already developed contraindications to the standard chemotherapy, olaparib (300 mg twice a day) was given as a last hope option. The patient demonstrated a “Lazarus response”: the performance status and the results of the biochemical tests went back to the norm within first two weeks of treatment. Positron emission tomography-computed tomography (PET-CT) was performed at one month after the start of olaparib therapy, and revealed complete metabolic response for all multiple metastatic lesions located in the liver, bones, small pelvis, lungs, mediastinum, retroperitoneum, etc. Cytotoxic therapy and poly ADP-ribose polymerase (PARP) inhibitors are known to have virtually identical mechanisms of tumor escape from the treatment, which are confined to the restoration of BRCA proficiency within cancer cells. The pronounced tumor response to the treatment in this patient can be attributed to the lack of recent exposure to standard cytotoxic treatment as well as to the inability of tumors with gross BRCA rearrangements to restore BRCA function via secondary mutation. This observation calls for comprehensive evaluation of PARP inhibitors in chemonaive patients with hereditary cancer.

## Introduction

BRCA1/2-driven hereditary cancers usually develop via somatic inactivation of the remaining allele of the involved gene. Therefore, while normal cells of the mutation carrier retain the capacity to cope with DNA damage, tumor cells become deficient for DNA repair by homologous recombination. As a result, BRCA1/2-associated cancers are selectively sensitive to certain DNA damaging agents, particularly cisplatin and mitomycin C, and generally demonstrate improved response rates to conventional chemotherapy. Furthermore, studies on pathogenesis of hereditary cancers led to the invention of a novel class of targeted drugs, whose mechanisms of tumor-specific action relies on the inhibition of poly ADP-ribose polymerase (PARP) [1].

PARP inhibitors (PARPi) already demonstrated substantial efficacy in cancers arising in BRCA1/2 mutation carriers, however, their clinical development is complicated due to some specific circumstances. Breast and ovarian cancers constitute the vast majority of BRCA1/2-associated malignancies. These types of tumors are highly chemosensitive, therefore, many standard treatment options exist for these oncological diseases. Due to ethical issues, PARPi could be subjected to clinical trials mainly when applied after the failure of conventional treatment schemes. However, while many of well-known targeted agents, such as inhibitors of mutated EGFR, BRAF or ALK, or antibodies against HER2 or EGFR, have little overlap with chemotherapy when considering their mechanisms of action and resistance, sequential administration of cytotoxic drugs and PARPi can be compromised by virtually identical routes of the tumor escape. For example, many BRCA1/2-associated cancers develop resistance to platinum compounds or PARPi via the gain of the second mutation, which is located in the vicinity of the germ-line BRCA1 alteration and restores the open reading frame of the gene [1,2]. In addition, the resistance of BRCA1-associated cancers to cisplatin or PARPi may involve skipping of the mutation-containing exon, use of alternative sites for translation and selection of BRCA1-heterozygous cells [3-5]. In any event, the cessation of the effect of both chemotherapy and PARPi usually involves restoration of BRCA1 proficiency in tumor cells. This explains the outcomes of clinical trials of PARPi, which demonstrated clinically significant but still moderate efficacy when applied after the cytotoxic treatment [6,7].

## Case presentation

Here we describe a BRCA2-mutated breast cancer (BC) patient, who received mainly endocrine therapy for the treatment of metastatic breast cancer and could not be subsequently treated by chemotherapy due to contraindications. She was administered with olaparib and demonstrated a “Lazarus response”.

**Figure 1:**
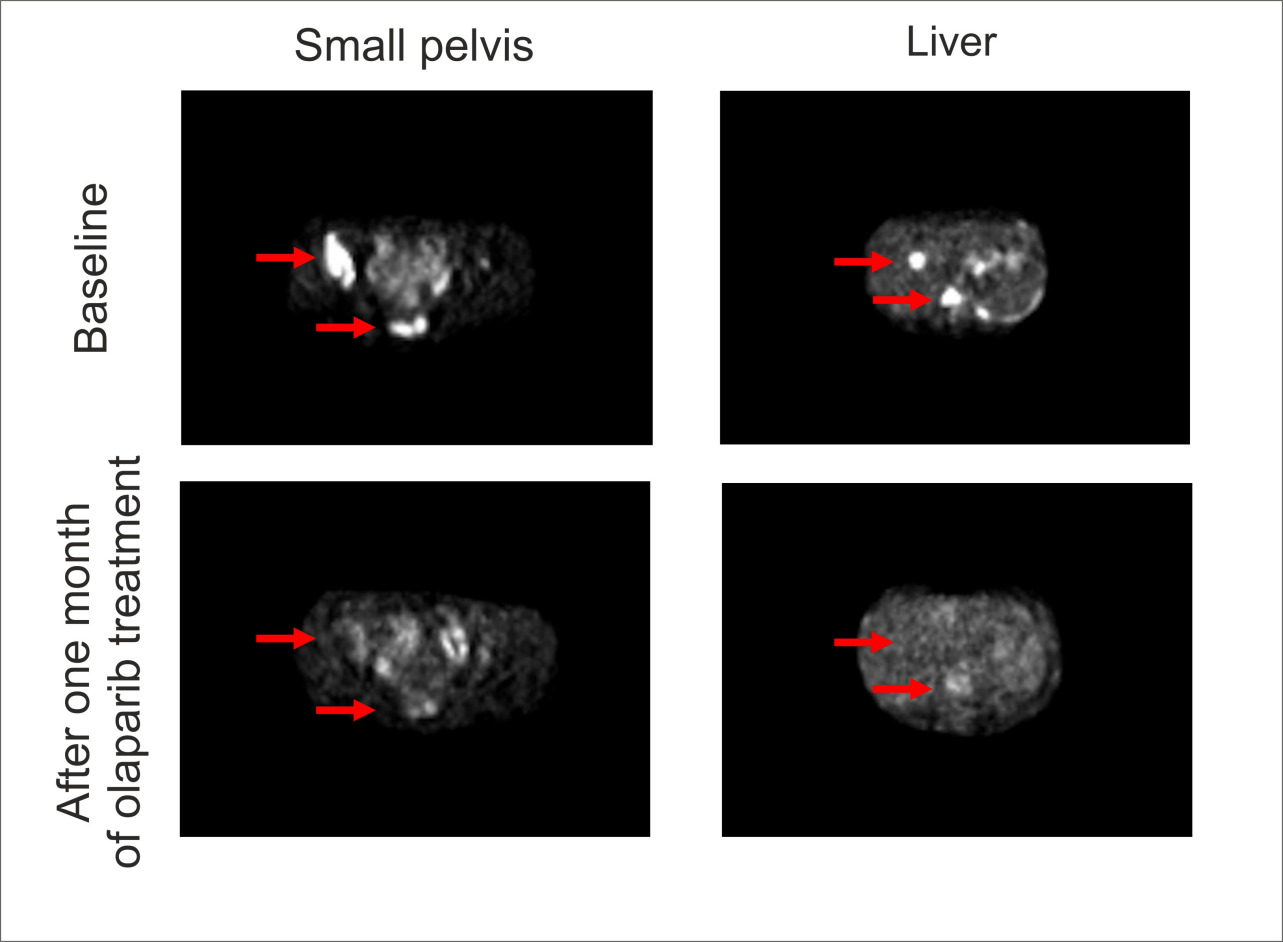
Administration of olaparib after failure of endocrine therapy causes complete metabolic response in BRCA2-driven breast cancer.

The patient was diagnosed with invasive ductal carcinomas of the left breast (T1N3M0; ER score: 8; PgR score: 0; HER2 score 0; Ki67 score 20%) while being 28 years of age. She was exposed to four cycles of standard neoadjuvant therapy using TAC combination (paclitaxel 175 mg/m^2^, doxorubicin 50 mg/m^2^, cyclophosphamide 600 mg/m^2^ every three weeks), which resulted in a partial clinical response. The analysis of tumor masses removed upon mastectomy revealed tumor down-staging to pT1N1M0 and preserved receptor status (ER+PgR-HER2-). TAC therapy was continued after the surgery for another four cycles. The patient experienced local tumor relapse right after the completion of adjuvant systemic treatment. The relapsed tumor lump was excised, and the radiological treatment was applied (36 Gy). Based on strong tumor positivity for estrogen receptors, tamoxifen was administered at a standard dose (20 mg per day). After seven months of tamoxifen treatment, the patient switched to exemestane (25 mg per day), and the ovariectomy was performed to allow the therapeutic aromatase inhibition. The treatment by exemestane appeared to be efficient for a period of two years, however, multiple bone, lymph node, and soft tissue metastases were subsequently revealed at a regular control examination. The biopsy of the tumor lump located within the soft tissues of anterior thoracic wall followed by morphological and immunohistochemical examination confirmed the relapse of breast cancer disease. Given that the prolonged effect of the aromatase inhibition and the features of metastatic spread corresponded well to the characteristics of hormone-sensitive BC, the continuing exemestane treatment was supplemented by mTOR inhibition (everolimus 10 mg per day) and bisphosphonate administration (clodronic acid 1600 mg per day). This therapy lasted for five months when the appearance of multiple new lesions in lungs, liver, and bones was observed. The patient was offered comprehensive BRCA1/2 germ-line testing, and the deletion of exons 1-18 of BRCA2 gene was revealed. The discussion on possible therapy options led to the choice of metronomic cyclophosphamide and methotrexate; this decision was based on the preference of the patient to receive oral drug formulations, the existing evidence for increased sensitivity of BRCA2-driven cancers to alkylating agents [1] and the prior history of prolonged disease control upon endocrine treatment. However, the condition of the patient deteriorated at a fulminant speed within the next 10 days. There was a significant worsening of the pain and increase of the levels of bilirubin (20.2 micromol/l), transaminases (ALT 591 units/l, AST 568 units/l) and creatinine (322 micromol/l). In addition, grade II anemia and grade III thrombocytopenia were observed. The patient, being at ECOG-3, was administered the infusion therapy aimed to improve the liver function as well as received a blood transfusion. Positron emission tomography-computed tomography (PET-CT) scan demonstrated multiple metabolically active metastatic lesions in the liver, bones, small pelvis, lungs, mediastinum, retroperitoneum, etc. Given the life-threatening condition of the patient and the contraindications to the standard chemotherapy, olaparib was administered at a dose 300 mg twice a day, starting in March, 2017; the reduced dose of the drug was chosen due to grade II liver insufficiency. Just after one week of the olaparib treatment, symptoms of hepatic and renal insufficiencies were resolved, and the results of biochemical and cellular analyses of the blood went back to the norm by the end of the second week of therapy. This was accompanied by full restoration of the performance status and the patient returned to work. PET-CT scan was performed at one month after the start of olaparib therapy and revealed the complete metabolic response (Figure 1). BRCA2-driven tumors are known to be highly sensitive to platinum compounds; furthermore, some instances of the cure of metastatic BRCA2-associated BC by high-dose therapy were described in the literature [1]. Based on these data, the patient was offered a consolidation therapy and received two cycles of carboplatin (AUC = 12, 1400 mg) in June 2017 and August 2017, respectively. Control PET-CT examination was performed in September 2017 and revealed some increase of standardized uptake value (SUV) in the sacrum (SUV = 10.2) and right acetabulum (SUV = 10.1). However, biopsy of the sacral bone revealed no tumor cells. In December 2017, the patient was diagnosed with the fracture of L3 vertebra, caused by an occasional awkward move. She was subjected to transpedicular osteosynthesis and laminectomy for the L3. Histological examination of excised lamina, which was a BC metastatic site at the time of the start of olaparib therapy, did not detect any tumor cells. While continuing PARPi treatment, the patient started to receive denosumab to maintain bone homeostasis.

## Discussion

BC patients relapsing after adjuvant TAC therapy have limited choices with regard to cytotoxic treatments. Current recommendations suggest to abstain from taxanes and anthracyclines in this situation, and consider single-agent therapy regimens [8]. Capecitabine could be regarded as a treatment of choice for this woman, given its promising activity towards ER-positive BC and convenience of administration of this oral drug [9]. Alternatively, therapy by carboplatin could be considered if making the emphasis on BRCA2-mutated background of this patient [1]. However, this female did not receive standard cycles of BC cytotoxic therapy but was given only metronomic cyclophosphamide and methotrexate for a period of 10 days. Lack of recent exposure to proper doses of cytotoxic drugs may be a reason for the unusual speed and the degree of the response to olaparib. Indeed, it is likely that tumor cells could not develop the escape pathways, which would overlap with PARPi resistance mechanisms. Interestingly, this patient carries an exceptionally rare type of BRCA1/2 mutation: it involves gross deletion located within BRCA2 gene, therefore the gene function cannot be restored by the secondary mutational event.

Recently published olaparib trial involved 59 BC patients with BRCA1/2 mutations, who received this PARPi as the front-line therapy for metastatic BC [10]. It is not commented in the report [10], what was the time interval between adjuvant treatment and olaparib administration, and whether any of the analyzed patients carried gross BRCA1/2 rearrangements; it is also not specified if some of these women omitted adjuvant cytotoxic therapy. Overall, Robson, et al. [10] demonstrated only a non-significant trend to improved responses to olaparib in the first-line versus second- or third-line settings. While comparing our observation with the study of Robson, et al. [10], it is essential to recognize that BRCA1- and BRCA2-driven tumors may differ with regard to mechanisms of drug sensitivity and resistance [1]. BRCA2-PARP synthetic lethality is known to be more pronounced than the one for BRCA1 gene [2]. Furthermore, restoration of the gene function via secondary mutation appears to be the only well-established route for tumor escape in BRCA2 mutation carriers; in contrast, there is a diversity of pathways associated with the adaptation to the treatment for BRCA1-related cancers, with some of them involving selection of preexisting BRCA1-proficient cells [5] or various non-genetic mechanisms [2]. Therefore, while tumors with small mutations in BRCA1 and BRCA2 genes may show similar natural history upon exposure to PARPi, the situation may become different for gross gene rearrangements, with BRCA2-associated cancers being more vulnerable to targeted therapeutic interventions.

## Conclusions

This study presents a BC patient with a large BRCA2 gene rearrangement, who demonstrated an unusually rapid and pronounced response to olaparib. The long-time interval between adjuvant chemotherapy and PARPi treatment as well as the inability of cells with gross BRCA2 deletions to restore the function of the gene appears to be the most plausible explanations for the exceptional sensitivity of this tumor to PARP inhibition. This observation calls for the comprehensive evaluation of PARPi in clinical trials involving chemonaive cancer patients.
